# Hydrogen Sulphide (H_2_S) Exposure Hazard Assessment: An Algorithm for Generating Exposure Index Based on Direct Instrument Readings

**DOI:** 10.1093/annweh/wxab047

**Published:** 2021-06-29

**Authors:** Åse Dalseth Austigard, Hans Thore Smedbold

**Affiliations:** 1 Department of Industrial Economics and Technology Management, NTNU—Norwegian University of Science and Technology, PO Box 8900, Torgarden, Trondheim N-7491, Norway; 2 Trondheim Municipality, Working Environment Office, PO Box 2300 Torgarden, N-7004 Trondheim, Norway; 3 Proactima AS, Richard Johnsensgt. 4, N-4021 Stavanger, Norway; 4 Department of Occupational Medicine, St Olav University Hospital, PO Box 3250, Torgarden, Trondheim N-7006, Norway

**Keywords:** exposure hazard assessment, exposure index, H_2_S, hydrogen sulphide, peak, time-weighted average, wastewater

## Abstract

**Objectives:**

Increased use of small affordable alarm sensors with logging or network capabilities has improved the ability to monitor exposure. The large datasets generated from these monitors calls for development of a computer algorithm to assess these data.

**Methods:**

We examined 88 time series of hydrogen sulphide (H_2_S) from wastewater works previously used for developing the exposure index. The time series covered 331 h, where 16 h had readings different from zero.

**Results:**

The developed algorithm reproduced the manual assessed index almost perfectly (linear regression *β* = 1.02, *R*^2^ = 0.97, *P* < 0.001). Time-weighted average (TWA) values of the 88 time series showed a mean value of 0.04 ppm (range 0.0–0.9). The mean index value was 18 (range 0–337), with a good linear fit (*β* = 0.002, *R*^2^ = 0.93, and *P* < 0.001). The index gave us a better resolution and basis for risk assessment than the TWA, and managed to combine evaluation of TWA and exceedance of ceiling value in one number.

**Conclusions:**

As long as peaks above ceiling value occur, we find alarm tools with an H_2_S sensor to be an essential personal protective equipment against H_2_S. The proposed method has been verified, and it removes some common human errors in graph evaluation. Use of the index is a possible way of quantifying risk level in exposure to H_2_S in one single number and provides better understanding of the risk of exposure, as it eases the analysis and evaluation of large numbers of time series.

**What’s important about this paper**

Gasses like hydrogen sulphide (H_2_S) have an acute effect on health and life during short exposures to high gas concentrations, but these peaks are not very visible on time-weighted average exposure measurements. Workers and employers need a tool to combine the risks of exposure duration and peak exposures. The algorithm proposed here is a such tool, and can convert large quantities of data from personal gas sensors into exposure index profiles. The index is demonstrated for H_2_S with peaks defined by exposures greater than 5 and 10 ppm, but can be applied to other gasses and with other definitions of peak exposures.

## Introduction

Exposure to hydrogen sulphide (H_2_S) is a main risk in work related to handling of wastewater. It releases from biological material that degrades by bacteria without access to oxygen. Wastewater can originate form households, agriculture, fishing industry, and in other industrial activities. H_2_S can be released coincidentally, as the gas pressure in sediments builds up, and when pressure drops on the sediments. Cleaning and flushing operations and disturbance of the sediments also release H_2_S. Some oil and gas fields contain large amounts of H_2_S. Sulphide ore melting operations and sulphur recovery units are other sources, and they might give high concentrations. We also find H_2_S in active geothermal areas of the world (Guidotti, 2015). It will not evaporate easily from cesspools, pits, tanks, and land depressions, as it is heavier than air.

In her review, Svendsen (2001) has described H_2_S as a sour gas that attacks mucous in the eyes and airway system by etching and can cause acute eye damages from a concentration of 20 parts per million (ppm), and acute lung effects from 250 ppm (Svendsen, 2001). Reaching 500–1000 ppm the gas is knocking a man unconscious without warning. H_2_S is special in the sense it has a low odour threshold of 0.01–0.3 ppm, but when exceeding 100 ppm, the smell disappears (Guidotti, 2015). This means that you do not have any smell as warning at levels of acute danger to health and life.

Studying the highly variable exposure is easier now that small and affordable alarm sensors with logging or network capabilities is available. In wastewater work, H_2_S exposure, together with monitoring of lower explosive limit, carbon monoxide, and oxygen, can be done with such sensors.

In epidemiology, the exposure under study is usually characterized by some indirect measure that is assumed to correlate with an adverse health outcome. For chronic diseases, the number of years in an occupation or an estimate of long-term average exposure summarized in a Job Exposure Matrix is often used (Rothman *et al.*, 2021). For transient or acute effects, other measures like peak exposure might be more relevant than traditional exposure statistics (Checkoway *et al.*, 2019; Virji and Kurth, 2020). We have previously proposed an exposure index for H_2_S that evaluates both intensity and frequency into one single number (Austigard *et al.*, 2018). Manual counting of peaks is time consuming when dealing with large amounts of data. Automatic counting might mean that nuances are overseen, but the alternative would usually mean that data are not collected or seen in detail at all.

In an outdoor or well ventilated area, H_2_S peaks are typically very steep and often last only some minutes. It is also normal that the exposure to H_2_S is restricted to some parts of the job. We should therefore expect zero exposure in large parts of every shift, and that exposure periods are separated by periods of no exposure between tasks or due to transportation to a new location. H_2_S peaks might exceed the 15-min short-term exposure limit (STEL) of 5 ppm in the USA (ACGIH, 2020), 10 ppm in European Union (ECHA, 2009), or the Norwegian ceiling value of 10 ppm (NLIA, 2020), without exceeding the threshold limit value of 1 ppm, or the 8-h occupational exposure limit (OEL) of 5 ppm in Norway and the EU.

The aim of this short communication is to describe and verify the automated algorithm by comparing its output with the original manual evaluation of the dataset, and to compare calculated index values to the time-weighted average (TWA) of a work day.

## Methods

The data used in this analysis were collected during 2013–2014 from municipal wastewater workers in three cities and four rural areas of Norway, including all parts of wastewater work, both large and small treatment plants, pipelines and cesspools. The data were collected with Dräger x-am 5000, Dräger PAC 7000, OdaLog L2/LL, and OdaLog LowRange. Measurements were started as the work shifts started and continued during the day, until their active sewage work ended and the workers came back on site. Measurements were distributed over all seasons, and flushing were recorded. For more details, we refer to an earlier publication (Austigard *et al.*, 2018).

We retrieved 88 of the original 93 time series from wastewater work used at developing the index. For the last five time series, we only had access to graphs. We wrote the algorithm in the syntax language of SPSS (IBM SPSS Statistics ver 26).

We identify exposure incidents by calculating a centred moving average (CMA) of 3 min. Tasks have to be separated by the same minimum time with unexposed work to be considered as a different task. This separation time was chosen from knowledge to wastewater work.

We identify distinct peaks within each task by first finding values being larger than both the previous and the next point, and second by the difference between the value and a 1-min CMA being minimum 25% higher. These two together define a peak. Number of peaks within the index specified intervals is counted. Number of data points within each index interval is counted and converted to time.

The seven index elements are number of peaks in four intervals (H_2_S_01_ = up to 1.0 ppm, H_2_S_1_ = 1.1–5.0 ppm, H_2_S_5_ = 5.1–10.0 ppm, and H_2_S_10_ = 10.1 ppm and higher), duration in two intervals (H_2_S_dur01_ = up to 5.0 ppm and H_2_S_dur5_ = above 5 ppm), and H_2_S_max_ = the maximum value of H_2_S in the measurement. The elements are weighted in accordance with the interval for gas concentration of the data point being evaluated. The index equation is presented in equation (1). The index (H_2_S_index_) is treated as dimensionless (Austigard *et al.*, 2018).


$$\begin{array}{ll} H_{2}S_{index}=\;\; & H_{2}S_{01}*0.1+H_{2}S_{dur01}*0.1+H_{2}S_{1}+H_{2}S_{5}*5\\ & +H_{2}S_{dur5}*5+H_{2}S_{10}*10+H_{2}S_{max} \end{array}$$ (1)

Readers are made aware that the exposure index published in 2018 (Austigard *et al*, 2018) contains a misspelling that is corrected in Heldal *et al.* (2019) and in this short communication (‘H_2_S_5_*5+’ is lacking the ‘5+’).

Full data information also allows calculation of average exposure level for the measurement time and TWA to compare with OEL. These values were not collected and analysed in the previous publications from this study (Austigard *et al.*, 2018; Heldal *et al.*, 2019).

## Results

Our dataset is 88 time series containing 107 785 data points, covering 331 h. A total of 9744 data points (32 h) have positive 3 min CMA, identifying 241 tasks. From these, 4689 data points (16 h) have positive readings. These exposed periods have a median exposure of 0.5 ppm (range 0.1–276 ppm) with geometric mean exposure of 1.8 ppm and geometric standard deviation 9.3 ppm. The mean task duration was 8 min, with median 5 min (range 3–78 min).

**Figure 1.**
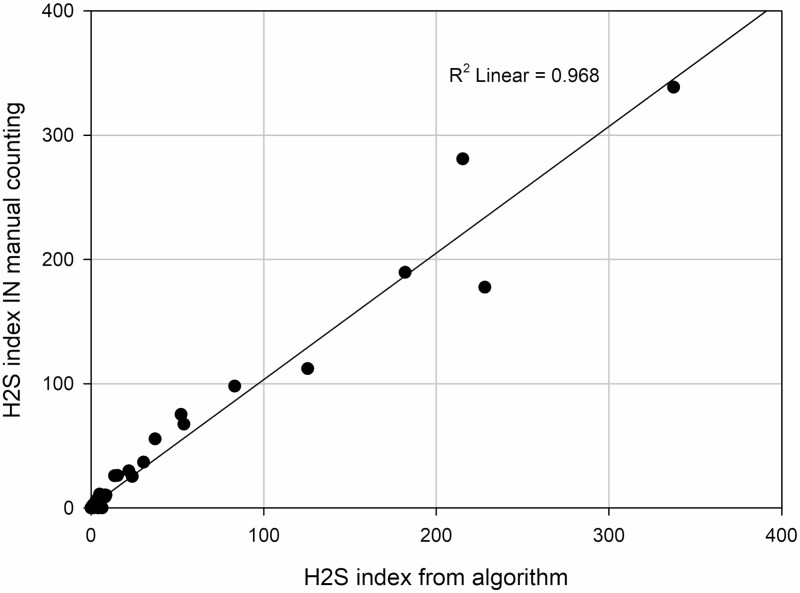
Plot of H_2_S index value from manual counting compared with algorithm calculation. The plot is marked with a linear regression line with equation *y* = 1.02*x + 1.27. Regression *R*^2^ = 0.97 with significance level *P* ≤ 0.001.

The total automatic index calculation corresponded to the index from manual counting, evaluated by linear regression (*y* = 1.02*x + 1.27 with *R*^2^ = 0.97, Fig. 1).

Kendall’s tau_b non-parametric correlation was 0.87. Calculations were made both on single elements and on the total index. All *R*^2^ values and all Kendall’s tau_b had significance level *P* < 0.001.

For the two outliers in Fig. 1, we explain most of the distance from the expected regression by inaccuracies in the manual estimation of time in different intervals and by the number of peaks counted above 10 ppm.

**Figure 2.**
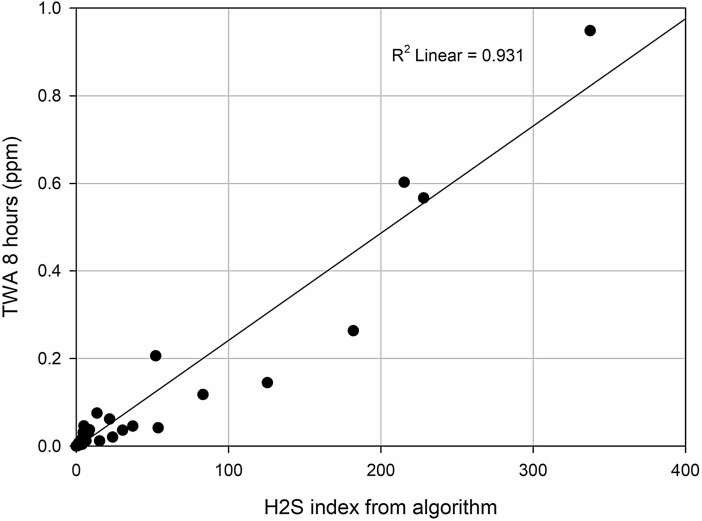
Plot of index to TWA for the 88 time series with linear regression line. Regression equation *y* = 0.002*x* − 0.003. Regression *R*^2^ = 0.93 with significance level *P* ≤ 0.001.

Analysis of the TWA values of the 88 time series gave a mean of 0.04 ppm (range 0.00–0.9), while the mean index value was 18 (range 0–337). The 10 time series with the highest peaks (range 10–276 ppm) had a mean and median TWA of 0.3 and 0.1 ppm, respectively, while the mean and median index were 85 and 52. None of the time series exceeds a TWA of 1.0 ppm. The TWA and index values for the time series are compared in Fig. 2 (*R*^2^ = 0.93).

## Discussion

In this short communication, we describe the main elements of the developed algorithm. The calculated index fitted well with the manual assessed data demonstrated with linear regression, and by the regression line going through the diagram slightly over 1:1 and 100:100 in Fig. 1. One explanation might be that in the original manual counting, a new peak was counted if the visual drop seemed deep, or if the measured level approached zero, regardless of the time it stayed there. In the algorithm, approaching zero is not enough to count a new peak. Every task might have multiple peaks, but at least one.

The manual counting was influenced by the scale of the printed graph. In our data, we saw this by two time series where the manual counting yielded zero, while automatic counting gave an index value about 2.5. The most plausible explanation for overlooking the peaks was the scale of the printout.

Three minutes separation time between tasks gives a period long enough to be sure that these are separate tasks, and at the same time that the average of really exposed periods are not too affected by the unexposed time. Using 5 min will camouflage different tasks, for example at inspections and cleaning at different pumping stations. Some of these have less than 5 min unexposed separation time. Separation time can be modified to fit other work patterns. Differentiating in tasks does not alter the index value, but in work with clearly separated operations during the day, it can be helpful in evaluation of tasks and locations.

The method of assessment can be used on all H_2_S data, if these are available in a CSV-list format, database, or spreadsheet, along with timestamps in regular logging intervals.

If the whole dataset of 88 time series were said to represent the true exposure, not a single day would be said to exceed 20% of OEL at 5 ppm, even though we had an extreme exposure above 200 ppm in one measurement. The 20% level of the OEL is interesting, as the standard EN 689 (CEN, 2019) states that even with only five measurements, one can expect 95% of the working days to conform with the OEL, if none of one’s measurements exceeds 20% of the OEL (CEN, 2019). At the revision of the Norwegian OEL in 2011, it was the TWA values available in the Norwegian national EXPO database that were used as the basis of exposure documentation (NLIA, 2011), and that was referred to at risk evaluation against OEL in different kinds of work. Data for ceiling value were not used. This supports the need of a different method of risk assessment, for example an exposure index. The OEL is the same over most of Europe, but most of the countries use a STEL value for 15 min, not a ceiling value. We chose the set points for differentiating peaks in intervals of the index according to these legislation levels. By construction it is however not restricted to use where these levels apply. The levels, 1, 5, and 10 ppm, all have relevance to biological effect (Svendsen, 2001), and if wanted, it is of course possible to adjust with other or more intervals and factors. This will however alter the results of the algorithm, and it will have to be re-tested.

The comparison of index value to 8-h TWA showed that the index varies linearly with the TWA. However, the index illustrated the increase in risk level in a better way, as it also includes assessment of the peak level and number of peaks. In our dataset, all time series that include peaks above ceiling level had an index value above 37, but the TWA values for these time series were as low as 0.04 ppm. The *β* value is 0.002. In Fig. 2, we can see this by the regression line crossing at (*x*, *y*) = (410, 1). This fits with the 95% confidence interval for the slope (0.002–0.003). We therefore find the index a better tool for risk assessment of H_2_S than comparing to OEL and ceiling value separately. The employers and employees need a single risk number to evaluate.

The TWA values that we found clearly show that it is the peaks that give the problematic exposure. The unpredictability of the exposure levels during the peaks makes us convinced that an alarm tool with an H_2_S sensor is an essential personal protective equipment in H_2_S and wastewater related work. It must be emphasized that sensor use must follow a clear routine on how to act if the alarm goes off. Far too often, a response to an alarm is to turn off the sensor while fulfilling the task, as the alarm is quite noisy. In that way you lose both the duration of exposure, the possibly high levels in your data, and most important: the intended protection given from the equipment.

At places and work where continuous surveillance is not possible, index values from some time series will give an indication where to prioritize measurements, adjustments, change in equipment and so on, in order to reduce exposure potential.

## Supplementary Material

wxab047_suppl_Supplementary_Material

## Data Availability

Data and algorithms will be made available at Norwegian Centre for Research Data, NSD (www.NSD.no). The algorithm will also be available online as a supplementary file.
