# Supplementary material for: Hydrogen Sulphide (H2S) Exposure Hazard Assessment: An Algorithm for Generating Exposure Index Based on Direct Instrument Readings
Source: Ann Work Expo Health. 2021 Jun 29;66(1):124–9. doi: 10.1093/annweh/wxab047 (PMC8751804; doi:10.1093/annweh/wxab047)
Supplement: wxab047_suppl_Supplementary_Material [file wxab047_suppl_supplementary_material.doc]

**Hydrogen sulphide (H2S) exposure hazard assessment: an algorithm for generating exposure index based on direct instrument readings**

Åse Dalseth Austigard ^1, 3^ (Corresponding author), Hans Thore Smedbold ^2, 4^

^1^ Department of Industrial Economics and Technology Management, NTNU - Norwegian University of Science and Technology, Trondheim, N-7491, Norway. E-mail: [ase.d.austigard@ntnu.no](mailto:ase.d.austigard@ntnu.no)

^2^ Proactima AS, Richard Johnsensgt. 4, N-4021 Stavanger, Norway. E-mail: [hans.thore.smedbold@proactima.com](mailto:hans.thore.smedbold@proactima.com)

^3^ Trondheim municipality, Working environment office, PO. box 2300 Torgarden, N-7004 Trondheim, Norway. E-mail: [ase-dalseth.austigard@trondheim.kommune.no](mailto:ase-dalseth.austigard@trondheim.kommune.no)

^4^ Department of Occupational Medicine, St Olav University Hospital, Trondheim, N-7006, Norway

**Syntax document – Algorithm for generation of index values from H2S data**

This document contain the SPSS syntax used in the generation of H2S index values, as presented in our short communication, “Hydrogen sulphide (H2S) exposure hazard assessment: an algorithm for generating exposure index based on direct instrument readings”.

* Encoding: UTF-8.

GET file='filepath\filename.sav'. */datafile with ID, Date, Time, measurement equipment.

DATASET NAME DataSet1 WINDOW=FRONT.

COMPUTE LOGID = ID.

SORT CASE by LOGID Date Time.

IF LOGID <> 0 Lnr = $casenum.

FORMATS Lnr (F8.0) LOGID (F4.0).

VARIABLE LABELS LOGID 'Standarized ID'

/Lnr 'Consecutive line number'.

EXECUTE.

** 2 * Exclude values considered as noise - below 0 ************************.

DATASET ACTIVATE DataSet1.

COMPUTE new_h2s=h2s.

COMPUTE H2S_negative=0.

IF (new_h2s < 0) H2S_negative=1.

EXECUTE.

IF h2s<0 new_h2s=0.

COMPUTE H2S_not_0=0.

IF (new_h2s <> 0) H2S_not_0=1.

VARIABLE LABELS new_h2s 'Measured level'

/H2S_not_0 'Tag: 1=not a 0-value'

/H2S_negative 'Tag: 1= negative value in original data'.

EXECUTE.

** 3 ** Time interval in minutes ** Identifies logging interval for each series and harmonizes**.

DATASET ACTIVATE DataSet1.

SORT CASES BY LOGID Lnr(A).

SPLIT FILE SEPARATE BY LOGID.

CREATE

/interval0=DIFF(Time 1).

SHIFT VALUES VARIABLE=Interval0 RESULT= Interval1 LEAD =1.

COMPUTE Interval2 = max(interval0, interval1) / 60.

FORMATS Interval2 (f3.2).

VARIABLE LABELS Interval2 'logging interval in min'.

EXECUTE.

SPLIT FILE OFF.

DELETE VARIABLES Interval0 Interval1.

** 3a * Cleaning up decimals of interval2 *****.

IF (interval2 < .25) interval2 = .167.

IF ((interval2 >0.2) and (interval2<0.3)) interval2 = 0.25.

IF (interval2 >5) interval2 = 10.

EXECUTE.

** 4 ** Identifies exposed time for each LOGID *********.

****** Calculates MA=Centralised Moving Average *******************************.

******* NB! Different syntax for different log intervals *******************************.

DATASET ACTIVATE DataSet1.

USE ALL.

SORT CASES BY ID Lnr.

SPLIT FILE SEPARATE BY LOGID.

IF (Interval2 = 10) Valid_10min = 1.

IF (Interval2 = 0.25) Valid_15sec = 1.

IF (Interval2 = 0.167) Valid_10sec =1.

FORMATS Valid_10min (f1.0) Valid_10sec (f1.0) Valid_15sec (f1.0).

VARIABLE LABELS Valid_10min 'Intermediate - Measurement interval 10 min. 0=no 1=yes'

/Valid_15sec 'Intermediate - Measurement interval 15 sec. 0=no 1=yes'

/Valid_10sec 'Intermediate - Measurement interval 10 sec. 0=no 1=yes'.

EXECUTE.

COMPUTE H2S_MA_10min = 0.

IF (Valid_10min = 1) H2S_MA_10min = new_h2s. /*No moving average calculated for 10 min mean.

CREATE H2S_MA_15sec = MA(new_h2s 12). /*MA 6+1+6 datapoint = 3 min 15 sec.

IF sysmis(Valid_15sec) H2S_MA_15sec = 0.

CREATE H2S_MA_10sec = MA(new_h2s 18). /*MA 9+1+9 datapoint = 3 min 15 sec.

IF sysmis(Valid_10sec) H2S_MA_10sec = 0.

EXECUTE.

COMPUTE H2S_MA_3min = H2S_MA_10min + H2S_MA_15sec + H2S_MA_10sec.

VARIABLE LABELS H2S_MA_10min 'Intermediate - Moving average if 10 min'

/H2S_MA_15sec 'Intermediate - Moving average if 3.25 min'

/H2S_MA_10sec 'Intermediate - Moving average if 3.17 min'

/H2S_MA_3min 'Moving average 3 min'.

EXECUTE.

SPLIT FILE Off.

EXECUTE.

** 5.0 ** Identifies peak. Differences according to logging interval.

DATASET ACTIVATE DataSet1.

SORT CASES BY ID Lnr.

SPLIT FILE SEPARATE BY ID.

CREATE

/H2S_lag1=LAG(new_H2S 1)

/H2S_lead1=LEAD(new_H2S 1)

/H2S_MA_7=MA(new_H2S 7)

/H2S_MA_5=MA(new_H2S 5).

VARIABLE LABELS H2S_lag1 'Intermediate - H2S Consecutive value'

/H2S_lead1 'Intermediate - H2S Previous value'

/H2S_MA_7 'Intermediate - Moving average for evaluation of peak if Interval2=0.17'

/H2S_MA_5 'Intermediate - Moving average for evaluation of peak if Interval2=0.25'.

EXECUTE.

COMPUTE Peak01 = 0.

COMPUTE Fraction = 0.

IF (H2S_MA_7>0 & Valid_10sec = 1) Fraction = new_H2S / H2S_MA_7. /*one minut CMA.

IF (H2S_MA_5>0 & Valid_15sec = 1) Fraction = new_H2S / H2S_MA_5. /*one minut CMA.

IF (new_H2S > H2S_lag1 and new_H2S >H2S_lead1) Peak01 =1. /*marks peaks.

IF (Valid_10min = 1 & Peak01 = 1) Fraction = 3.

IF (Fraction >= 1.25 and Peak01 >= 1) Peak01 = 2.

/*Fraction 1.25 means at least 25% above CMA. Fraction >=2 means the peak is at least 100% over background (MA 1min+1 datapoint).

FORMATS Peak01 (f1.0).

VARIABLE LABELS Peak01 'Peak: 0=no 1=higher than both neighbours 2=yes'

/Fraction 'Relative level to CMA'.

SPLIT FILE OFF.

EXECUTE.

** 6 ** Identifies and count insidences of positiv MA (=task) .

DATASET ACTIVATE DataSet1.

USE ALL.

SORT CASES BY LOGID Lnr(A).

SPLIT FILE SEPARATE BY LOGID.

COMPUTE exposed_task = 0.

IF (H2S_MA_3min > 0) exposed_task = 1.

FORMATS exposed_task (f1.0).

EXECUTE.

COMPUTE task = 0. /*Only the first datapoint in each task is marked.

CREATE

/task=DIFF(exposed_task 1).

IF (task= -1) task = 0.

EXECUTE.

COMPUTE task_id =0. /*Each datapoint in the same task is marked with the same number.

CREATE

/task_id=CSUM(task).

IF (exposed_task = 0) task_id = 0.

EXECUTE.

SPLIT FILE OFF.

EXECUTE.

** 6a * Makes an unique job ID: task_id2. no decimals means unexposed data ***.

COMPUTE task_id2 = 0.

COMPUTE task_id2 = logid+task_id/100.

EXECUTE.

VARIABLE LABELS exposed_task 'Exposed task: 0=no, 1=yes'

/task 'Marks start point of tasks for counting'

/task_id 'Tasknumber within measurement'

/task_id2 'Identifikation number measurement and task'.

** 6b ** Finds MAX H2S for each task. Adds it to the dataset ******************.

DATASET ACTIVATE DataSet1.

USE ALL.

SORT CASES BY task_id2 Lnr.

AGGREGATE

/OUTFILE=* MODE=ADDVARIABLES OVERWRITEVARS=YES

/PRESORTED

/BREAK=task_id2

/h2s_task_max=MAX(new_h2s) /*Inserts and updates level each time the data gives a higher H2S-level within a task-id2.

/N_BREAK=N. /*Count the number of datapoints in each measurement in each task_ID2.

COMPUTE Time_taskID2 =N_break*Interval2.

VARIABLE LABELS h2s_task_max 'Maximum H2S value in task'

/N_BREAK 'Intermediate: Number of datapoints in task'

/Time_taskid2 'Duration of task in min' .

EXECUTE.

** 7 ** Finds level of exposure in intervals according to published index ****.

DATASET ACTIVATE DataSet1.

SORT CASES BY task_id2.

SPLIT FILE SEPARATE BY LOGID.

COMPUTE n_h2s01 = 0.

COMPUTE n_h2s1 = 0.

COMPUTE n_h2s5 = 0.

COMPUTE n_h2s10 =0.

COMPUTE n1_h2s01 = 0.

COMPUTE n1_h2s5 = 0.

FORMATS n_h2s01(f1.0) n_h2s1(f1.0) n_h2s5(f1.0) n_h2s10(f1.0) n1_h2s01(f1.0) n1_h2s5(f1.0).

VARIABLE LABELS n_h2s01 'Intermediate: new_H2S is within interval 0 - 1.0 0=no 1=yes'

/n_h2s1 'Intermediate: new_H2S is within interval 1.1-5.0 0=no 1=yes'

/n_h2s5 'Intermediate: new_H2S is within interval 5.1 - 10.0 0=no 1=yes'

/n_h2s10 'Intermediate: new_H2S is within interval >10 0=no 1=yes'

/n1_h2s01 'Intermediate:Datapoint in LOGID that is =<5.0 ppm. 0=no 1=yes'

/n1_h2s5 'Intermediate: Datapoint in LOGID that is >5.0 ppm. 0=no 1=yes'.

** 7a * Gives value 1 if new_H2S is in the current interval, else 0 ****.

COUNT n_H2S01=new_h2s(0.001 thru 1).

COUNT n_H2S1=new_h2s(1.1 thru 5).

COUNT n_H2S5=new_h2s(5.01 thru 10).

COUNT n_H2S10=new_h2s(10.1 thru 1000).

COUNT n1_H2S01=new_H2S(0.001 thru 5).

COUNT n1_H2S5=new_H2S(5.01 thru 1000).

EXECUTE.

** 7b * Gives value 1 if peak in current interval, else 0 ****.

COMPUTE h2s01_count0 =0.

COMPUTE h2s1_count0 =0.

COMPUTE h2s5_count0 =0.

COMPUTE h2s10_count0 =0.

FORMATS h2s01_count0(f1.0) h2s1_count0(f1.0) h2s5_count0(f1.0) h2s10_count0(f1.0).

VARIABLE LABELS h2s01_count0 'Tag of peak in interval 0-1.0. 0=no 1=yes'

/h2s1_count0 'Tag of peak in interval 1.1-5.0ppm. 0=no 1=yes'

/h2s5_count0 'Tag of peak in interval 5.1-10.0. 0=no 1=yes'

/h2s10_count0 'Tag of peak in interval >10ppm. 0=no 1=yes'.

IF (n_H2S01=1 & Peak01=2) h2s01_count0=1.

IF (n_H2S1=1 & Peak01=2) h2s1_count0=1.

IF (n_H2S5=1 & Peak01=2) h2s5_count0=1.

IF (n_H2S10=1 & Peak01=2) h2s10_count0=1.

EXECUTE.

** 7c * Finds number of counts for each element of the index for each LOGID ***.

SPLIT FILE off.

COMPUTE h2s01_count =0.

COMPUTE h2s1_count =0.

COMPUTE h2s5_count =0.

COMPUTE h2s10_count =0.

FORMATS h2s01_count(f1.0) h2s1_count(f1.0) h2s5_count(f1.0) h2s10_count(f1.0).

VARIABLE LABELS h2s01_count 'Number of peaks in interval per LogID 0-1.0'

/h2s1_count 'Number of peaks in interval per LogID 1.1-5.0ppm'

/h2s5_count 'Number of peaks in interval per LogID 5.1-10.0'

/h2s10_count 'Number of peaks in interval per LogID >10ppm'.

EXECUTE.

AGGREGATE

/OUTFILE=* MODE=ADDVARIABLES OVERWRITE = yes

/BREAK=LOGID

/h2s01_count =sum(h2s01_count0)

/h2s1_count = sum(h2s1_count0)

/h2s5_count = sum(h2s5_count0)

/h2s10_count = sum(h2s10_count0)

/h2s_logid_max = max(new_h2s)

/h2s_logid_mean = mean(new_h2s)

/N_logid =N.

EXECUTE.

AGGREGATE

/OUTFILE=* MODE=ADDVARIABLES OVERWRITE = yes

/BREAK=logid

/h2s01_count1 =sum(n1_h2s01)

/h2s5_count1 = sum(n1_h2s5).

EXECUTE.

FORMATS H2S_logid_max (F4.2) H2S_logid_mean (F4.2).

VARIABLE LABELS h2s01_count 'Number of datapoints per LOGID in interval<= 1.0 ppm'

/h2s1_count 'Number of datapoints per LOGID in interval1.1 -5.0 ppm'

/h2s5_count 'Number of datapoints per LOGID in interval 5.1 - 10.0 ppm'

/h2s10_count 'Number of datapoints per LOGID in interval>10.0 ppm'

/h2s_logid_max 'Maximum H2S-level in LOGID'

/h2s_logid_mean 'Mean H2S-level in LOGID'

/N_logid 'Number of measurement points per LOGID'

/h2s01_count1 'Number of measurement points per LOGID in interval <=5.0 ppm'

/h2s5_count1 'Number of measurement points per LOGID in interval >5.0 ppm'.

DATASET ACTIVATE DataSet1.

Filter OFF.

Use ALL.

COMPUTE Time_positive = 0.

COMPUTE Time_pos01 = h2s01_count1*interval2.

COMPUTE Time_pos5 = h2s5_count1*interval2.

COMPUTE Time_positive = Time_pos01 + Time_pos5.

VARIABLE LABELS Time_positive 'Time with positve data'

/Time_pos01 'Duration in min <= 5 ppm'

/Time_pos5 'Duration in min > 5 ppm'.

EXECUTE.

** 8 ** Calculates H2S_index ************************************************.

DATASET ACTIVATE DataSet1.

IF (interval2 = 0.167) interval2=1/6.

EXECUTE.

SORT CASES BY task_id2 LOGID.

SPLIT FILE SEPARATE BY LOGID.

COMPUTE n_h2s_index = 0.

COMPUTE n_h2s_index_1 = h2s01_count*0.1.

COMPUTE n_h2s_index_2 = h2s1_count.

COMPUTE n_h2s_index_3 = h2s5_count*5.

COMPUTE n_h2s_index_4 = h2s10_count*10.

COMPUTE n_h2s_index_5 = h2s_logid_max.

COMPUTE n_h2s_index_6 = Time_pos01*0.1.

COMPUTE n_h2s_index_7 = Time_pos5*5.

COMPUTE n_h2s_index =n_h2s_index_1 + n_h2s_index_2 + n_h2s_index_3 + n_h2s_index_4 + n_h2s_index_5 + n_h2s_index_6 + n_h2s_index_7.

VARIABLE LABELS n_h2s_index 'Index value for measurement'

/n_h2s_index_1 'Index element: number of peaks=<1 ppm'

/n_h2s_index_2 'Index element: number of peaks 1.1 - 5.0 ppm'

/n_h2s_index_3 'Index element: number of peaks 5.1 - 10.0 ppm'

/n_h2s_index_4 'Index element: number of peaks >10 ppm'

/n_h2s_index_5 'Index element: maximum H2S level in measurement'

/n_h2s_index_6 'Index element: Time =< 5 ppm'

/n_h2s_index_7 'Index element: Time > 5 ppm'.

SPLIT FILE OFF.

EXECUTE.

** End of syntax to convert measurement data to index value *****************

*SAVE OUTFILE='filepath\filename2.sav'

/COMPRESSED.
